# Analysis of medication-induced xerostomia in elderly Japanese patients

**DOI:** 10.1007/s00784-021-04182-2

**Published:** 2021-09-28

**Authors:** Hitomi Ono Minagi, Yoshie Yamanaka, Kanji Nohara, Kazuki Ikai, Takayoshi Sakai

**Affiliations:** 1 Department of Cytology and Histology, Dentistry and Pharmaceutical Sciences, Okayama University Graduate School of Medicine, Okayama, Japan; 2 Department of Oral-Facial Disorders, Osaka University Graduate School of Dentistry, Osaka, Japan; 3 Center for the Special Needs Dentistry, Okayama University Hospital, Okayama, Japan

**Keywords:** Xerostomia, Hyposalivation, Medication, Drug, Saxon test, Mouth moisture test

## Abstract

**Objectives:**

To determine the general condition of elderly xerostomia patients, we collected their background and medication data in order to potentially treat their xerostomia. It is critical to identify the drugs causing xerostomia in elderly patients. A total of 521 patients who were examined at the Xerostomia Clinic of Osaka University Dental Hospital were included in the study. We obtained patients’ data on age, sex, number of primary illnesses, Saxon test scores, oral moisture test, subjective symptoms, and drug types from their clinical records.

**Results:**

The mean age of the patients was 65.2 ± 13.3 years. Although all patients exhibited xerostomia symptoms, there were a lot of patients without hyposalivation. With respect to medication, each elderly xerostomia patient took an average of 6.8 ± 4.4 medicines. A total of 26.1% of patients in their 70 s took more than ten number of drugs. In addition, the number of frequently used medication medicine was different between elderly and young patients. Most of the medicines had xerostomia as a side effect in medical package inserts. Moreover, the quantity of salivation significantly decreased in patients who took more than seven drugs in comparison with the patients who did not take medicine.

**Conclusions:**

As patients age, the number of medications they take tends to increase, subsequently increasing their risk of xerostomia. For the health of the patients, it is critical that an accurate diagnosis is made.

**Clinical relevance:**

To establish therapeutic strategies for treatment of xerostomia, this study provides new and important information that will help in the development of xerostomia medical treatment.

**Supplementary Information:**

The online version contains supplementary material available at 10.1007/s00784-021-04182-2.

## Introduction

Xerostomia leads to various life-disrupting effects [1], because saliva has several physiological functions. Salivary gland diseases such as Sjögren’s syndrome (SS) and iatrogenic cause such as aftereffects of radiation therapy cause serious hypofunction. But there are many cases of xerostomia caused by multiple factors, and xerostomia can sometimes be caused by an underlying problem or medical condition [2–4]. It is known that xerostomia is the abnormal reduction of saliva and can be a symptom of certain diseases or be an adverse effect of certain medications. Furthermore, one common side effect of medications is xerostomia, which can significantly affect the patients’ overall health condition [5, 6]. Several reports show that a wide range of drugs can result in xerostomia. For drug-induced xerostomia, using the lowest effective dose or switching to an alternative medication may help. However, there are few considerations regarding the underlying situation and the patients’ intake of medicines for xerostomia [7, 8]. Xerostomia is a common complaint observed in the elderly who often complain of oral dryness as a result of underlying diseases and as a side effect of taking drugs [9]. Because the elderly often take several medications simultaneously, they increase the possibility of drug-related xerostomia [2]. However, there are only a few studies that examine the underlying situation of taking medicine among xerostomia patients. Hence, this study undertook a detailed analysis of the medications in xerostomia patients.

The correspondence methods for xerostomia patients vary among healthcare workers because causes of xerostomia are multifactorial. Existing therapeutic strategies are not definitive or effective for xerostomia patients^2^. To potentially develop possible treatment methods for xerostomia, collecting xerostomia patients’ basic data is significantly important. Basic data promote the establishment of the diagnostic methods and the development of possible treatment methods for xerostomia. In Osaka University Dental Hospital, inspection, diagnosis, and treatment for xerostomia have been performed. In this study, we collected the basic data of patients in the outpatient department and performed clinical examination. This study aims to examine the medication for the purpose of providing new treatment strategies for xerostomia patients.

## Subjects and methods

### Study design

A total of 521 adult patients were recruited from Osaka University Dental Hospital, Japan, between January 2013 and December 2017. All patients experienced xerostomia symptoms (such as oral dryness, glossalgia, and sticky saliva). Patients’ data regarding age, sex, primary illness, and drugs was obtained from their clinical records. Only prescribed medicines were included in this study, and over-the-counter drugs and supplements were excluded. Drug efficiency was classified using the Japan standard product classification of drugs that was established in the Ministry of Internal Affairs and Communications. To minimize identification errors, the investigator who took the measurements was blinded to the patients’ identity.

### Saxon test

The Saxon test measures the amount of stimulated saliva secretion^10^. The patients chewed two sterile gauzes (Hakujuji Sterauze) for 2 min. Subsequently, saliva secretion was calculated from the change in weight of the gauze before and after chewing. Saliva weight < 2.00 (g/2 min) and ≥ 2.00 (g/ 2 min) were considered positive and negative, respectively.

### Moisture test

#### Oral moisture test with Mucus®

The oral moisture levels of the lingual mucosa, dorsum of the tongue were measured using an oral moisture-checking device (Mucus®, LIFE Co.) [11]. The measurements were repeated three times, and the median value was chosen. Scores ≤ 27.9 were categorized under xerostomia^11^.

### The visual analogue scale (VAS)

Patients evaluated their symptoms of oral dryness on the VAS scale. VAS can be compared to other linear scales by every individual.

### Statistical analysis

The Statistical Package for the Social Sciences (SPSS) version 23 (SPSS Inc.) was used for statistical analysis. A multiple regression analysis of factors affecting the Saxon test score was performed. A paired *t*-test was used to compare the average values of the continuous variables. A chi-squared test and Bonferroni were used to compare the proportions of the categorical variables (such as saliva expression) between the groups. The data were presented as the mean values and standard deviation. Spearman’s rank correlation coefficient was used to determine the association between age and number of drugs.

## Results

### Patients’ baseline characteristics

**Fig. 1:**
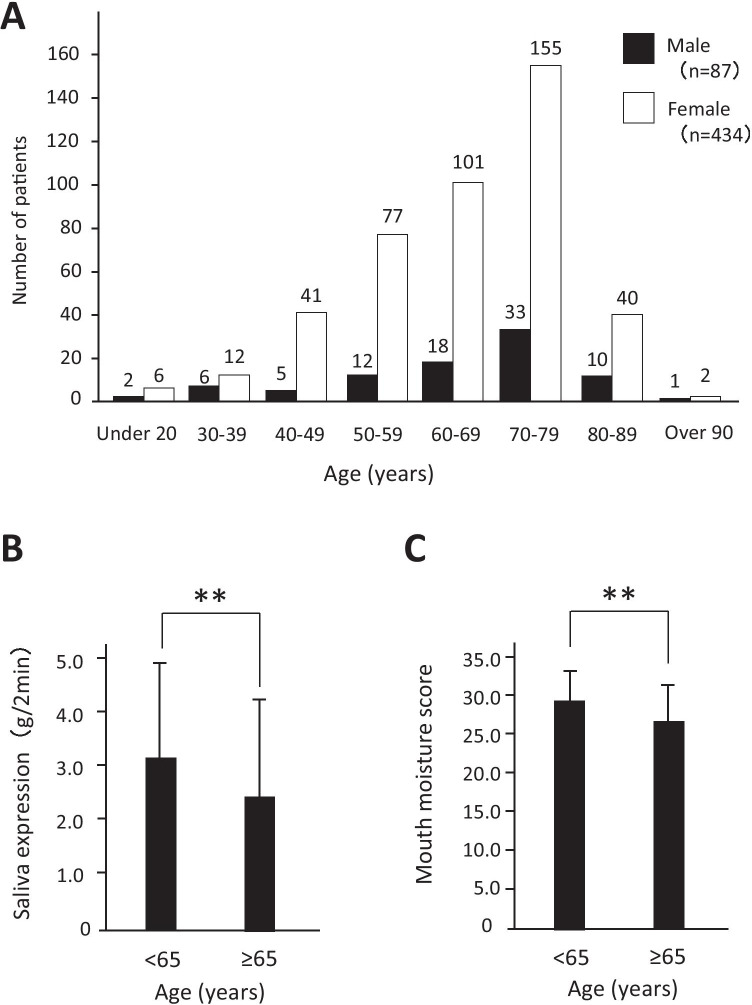
Patients’ baseline characteristics. This graph shows the number of patients with age distribution by gender. The vertical line shows the number of patients, and the horizontal line shows their ages (**A**). Score of Saxon test between < 65 years and ≥ 65 years patients. Saxon test scores < 2.00 (g/2 min) and ≥ 2.00 (g/2 min) are positive and negative for hyposalivation, respectively (**B**). Score of mouth moisture test between < 65 years and ≥ 65 years patients (**C**)

A total of 521 patients (male:female, 87:434) were recruited (Table 1). The number of female patients was more than five times as many as the male patients. The mean age of the patients was 65.2 ± 13.3 (range, 20–91 years) years, and the patients in this study were predominantly in their 70 s (Fig. 1A). The percentage of patients over 65 was 60.7%, and most of the patients were elderly females. The Saxon test was considered an inspection method for salivary gland hypofunction. The mean of the Saxon test was 2.74 ± 1.85 (g/2 min) (range, 0.02–12.51). In the *t*-test, the Saxon test scores were significantly lower among the elderly (Fig. 1B). Furthermore, the elderly scored significantly lower in the oral moisture test than the young patients (Fig. 1C). It is evident that elderly patients exhibited more serious hyposalivation than young patients from two inspections. A total of 65.3% of patients were negatively diagnosed with hyposalivation using the Saxon test. Although all patients experienced xerostomia symptoms, most of the patients had negative scores on the Saxon test. The percentage of patients with no primary disease was 14.6%, and most of the xerostomia patients had several other diseases (data not shown). There were many patients who had primary diseases in the following order: high blood pressure, eye diseases, and metabolic diseases. Diseases of the musculoskeletal system and connective tissue resulted in dry eye. A total of 42 (8.1%) of the patient population patients were diagnosed with SS. Although there were patients who were diagnosed with SS, xerostomia patients who were not diagnosed with SS often had dry eye.

**Table 1:** Patients’ baseline characteristics

	Total (*n* = 521)				< 65 (*n* = 205)				≥ 65 (*n* = 316)			
	Mean	SD	Minimum	Maximum	Mean	SD	Minimum	Maximum	Mean	SD	Minimum	Maximum
Age (years)	65.2	13.3	20	91	51.7	9.7	20	64	74.1	5.8	65	91
Male:female	87:434				29:176				58:258			
Saxon test (g/2 min)	2.74	1.85	0.02	12.51	3.06	1.9	0.05	11.25	2.53	1.78	0.02	12.61
Moisture	27.3	4.3	0.6	34.0	28	3.3	5.5	33.7	26.9	4.8	0.6	34.0
Medication	5.6	4.6	0	26	3.9	4.3	0	21	6.8	4.4	0	26
Primary illness^a^	2.5	2.0	0	11	2.0	2.0	0	11	2.8	1.9	0	10

^a^Primary illness is the number of primary illnesses

### Examination of medications

**Fig. 2:**
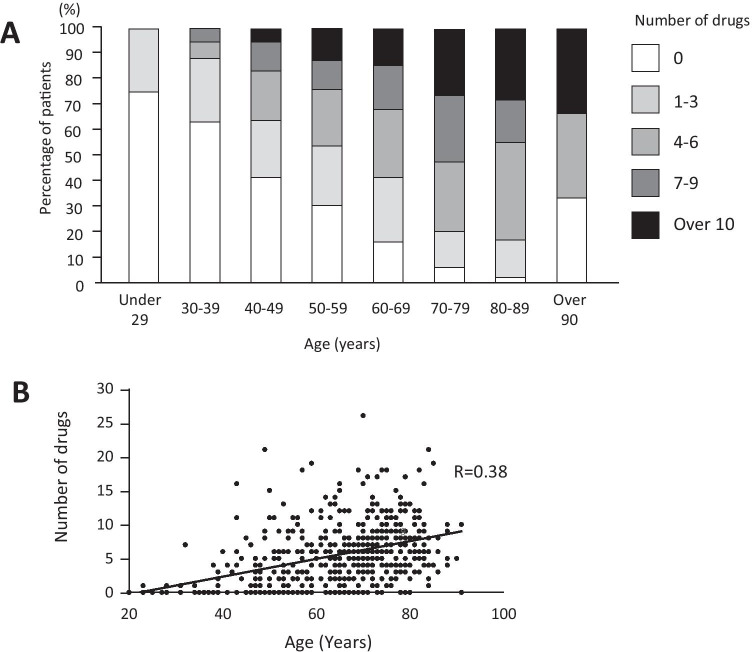
Investigation of remedy medication. This graph shows the age-specific number of taking medicine. With increasing age, the number of individuals taking medicine increased (**A**). The graph shows the correlation of reduction of age and the number of drugs (**B**)

**Fig. 3:**
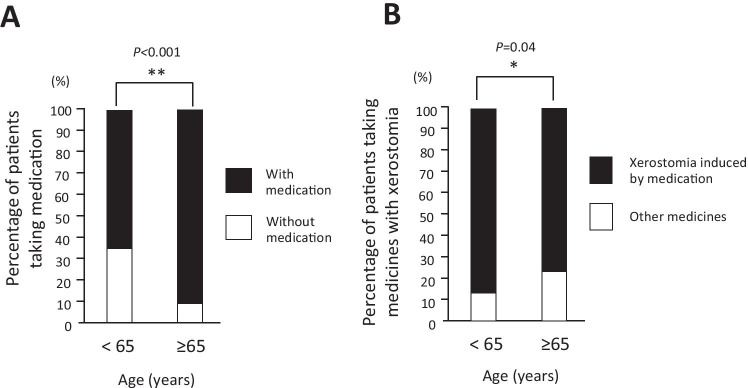
Situation of elderly person and remedy medication. The graph shows the ratio of patients with/without mediation. 65.9% in < 65 years patients and 92.4% in ≥ 65 years patients were treated with medication each (**A**). The graph shows the ratio of patients with drugs that have/do not have xerostomia as side effect. 86.7% in < 65 years medication patients and 77.7% in ≥ 65 years medication patients took at least one number of medicine with xerostomia each (**B**)

We examined the medication of the patients to reveal the underlying situations of patients who had xerostomia symptoms. To reveal the general condition of xerostomia elderly patients, we collected their background and medication data to potentially treat their xerostomia. Patients who took several medicines were assessed. Unfortunately, medicines prescribed to treat one disease can sometimes cause unwanted side effects; in this case, xerostomia. We summed up the number of medicines that were taken per person. The average number of medicines that were taken per person was 5.6 ± 4.6 (range, 0–21) drugs (Table 1). We investigated the age-specific number of patients taking several medicines (Fig. 2A). Although 75% of patients in their 20 s did not take medicines, only a limited number of patients were observed. To clarify the relationship between age and the number of medicines, we used the Pearson correlation coefficient (Fig. 2B). There was a slight correlation between xerostomia patients’ age and the number of medications takes. With increasing age, the number of drugs taken per person also increased. In the chi-squared test, the ratio of elderly patients with medication was significantly lower than in the younger group (Fig. 3A). A total of 81% of patients took several medicines. Using the efficacy classification, medicines for peptic ulcers, sedative and anti-anxiety agents, and medicines for psychoneurosis were significantly assessed. Because high blood pressure was the most prevalent condition the patients had in this investigation, several therapeutic drugs for high blood pressure were available. Furthermore, we classified medicine in elderly patients by generic names to analyze in detail (Table 2). Magnesium oxide, which is known as a laxative, was the most frequent, followed by etizolam and zolpidem tartrate. Several medicines with xerostomia as the side effect were available at high frequency (App-Table 1). Significant differences were observed between the two groups in magnesium oxide and amlodipine besilate. The ratio of medicine with xerostomia as the side effect was noted in the medical package inserts. A majority of the medicines had xerostomia as the side effect when we removed the drugs with unknown frequency. For example, Toviaz® (fesoterodine fumarate) was contraindicated in patients with urinary retention and gastric retention, and side effect incidence of Toviaz® was 40.9%. Additionally, xerostomia incidences of Bup-4® (propiverine hydrochloride) and Zyprexa® (olanzapine) were 20.2% and 11.8%, respectively. We investigated the side effects of xerostomia based on medical package inserts, and xerostomia as a side effect of medicines was observed in several patients. Because the values of the appearance frequency vary, the frequency listed in the medical package inserts refers to various papers. According to the classification of generic name, the largest was magnesium oxide followed in order by etizolam, zolpidem tartrate, and amlodipine besylate. Drug prescription rate is the ratio of patients taking the drug divided by all patients. Finally, 86.7% of young medication patients and 77.7% of elderly medication patients took at least one medicine with xerostomia each (Fig. 3B). There were fewer patients younger than 65 years in the ratio of patients taking medicine. This study revealed that there were many patients who took medicine with xerostomia as a possible side effect.

**Table 2:** The drugs that patients take frequently in elderly patients

Generic name	Drug name	Frequency of xerostomia (%)	Medicine	Drug prescription rates (%)		
				< 65	≥ 65	*p*-value
Magnesium oxide	Magnesium oxide®	Unknown	Antacids and laxatives	9.3	19.6	0.001
Amlodipine besilate	Norvasc®	< 0.1	Antihypertensive/angina	12.7	16.8	0.0004
Etizolam	Depas®	< 0.1–5	Tranquilizer	14.1	13.9	0.96
Zolpidem tartrate	Myslee®	< 0.1–5	Hypnotic	12.7	12.7	0.46
Brotizolam	Lendormin®	< 0.1	Sleep inducer	10.7	12.7	0.49
Atorvastatin calcium hydrate	Lipitor®	< 0.1	HMG-CoA reductase inhibitor	9.3	12.3	0.27
Lansoprazole	Takepron®	< 0.1–5	Proton pump inhibitor	9.8	12	0.41

**Fig. 4:**
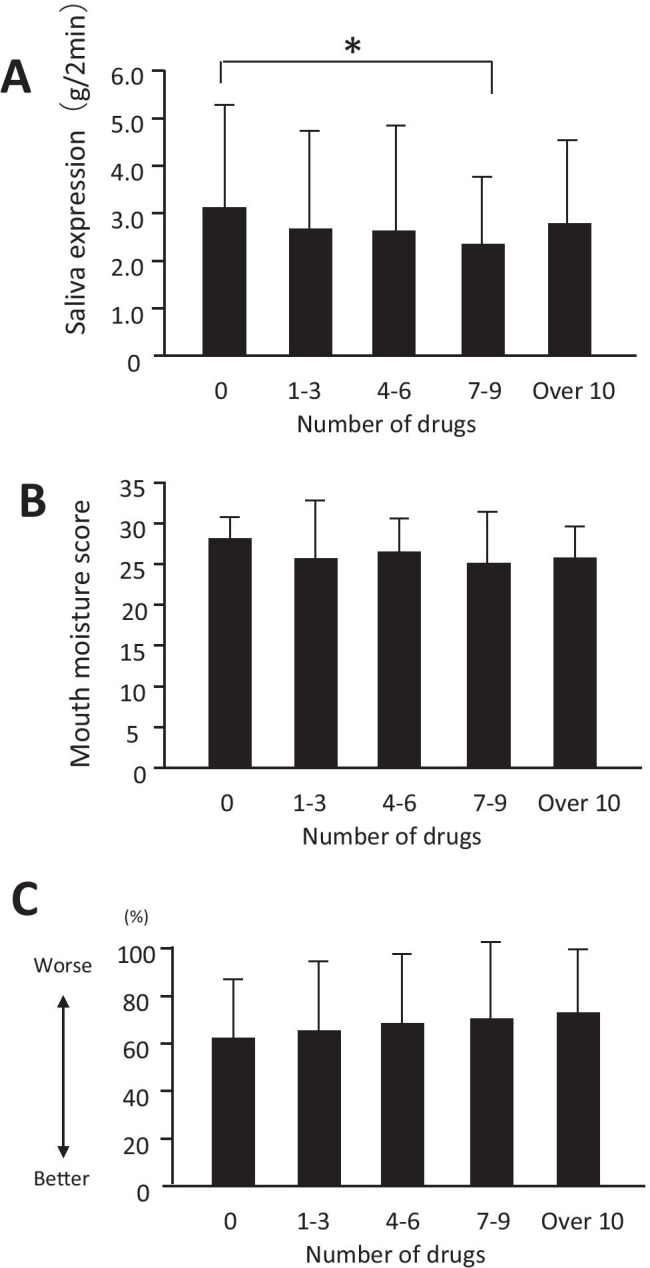
Difference of the symptom by the quantity of taking medicine. The bar graph shows the number of remedy medication with scores of Saxon tests (**A**). The bar graph shows the number of remedy medication with scores of moisture of mouth (**B**). Graph comparing visual analogue scale (VAS) scores. The bar graph shows the number of remedy medication with scores of VAS. Mean symptom scores for dry mouth on the visual analogue scale for patients. Patients feel more serious for xerostomia as the score of the VAS is bigger (**C**). *P < 0.05 by the Bonferroni method

We revealed the relationships between the numbers of drugs and objective symptoms to clarify the actual situation of the medication as the side effect. Patients who took 7–9 number of drugs showed a lower Saxon test score than patients who did have medication (Fig. 4A). Similarly, we analyzed using the oral moisture test, but there were no meaningful differences (Fig. 4B). The VAS was employed to form a clinical opinion of each patient’s subjective symptoms. It is thought that patients feel more serious for xerostomia as the score of the VAS is bigger. Although meaningful differences were not evident between the number of drugs and patient’s objective symptoms, the number of drugs was (Fig. 4C).

Finally, the multiple regression analysis was performed to reveal the factors that affecting hyposalivation directly. We examined the Saxon test scores as dependent variable and age (years), sex (male or female), primary illness (the number of primary illness), and medication (number of drugs) as dependent variables. The meaningful probability was set as *p* = 0.001, and the decision coefficient was *R*^2^ = 0.061. The independent variables age and sex were extracted as significant factors affecting the Saxon test score (Table 3). Moreover, saliva expression of Saxon test and moisture score were examined in patients without medication. Hyposalivation that caused xerostomia in elderly patients was significantly decreased compared with younger patients without medication (App-Fig. 1).

**Table 3:** Related factors of patients’ baseline characteristics with scores of Saxon test

coefficient	Partial regression	Standard partial regression coefficient	*p-*value	Lower 95%	Upper 95%
Constant	6.00		0.00	10.842	7.092
Sex	− 0.905	− 0.183	0.00	− 4.301	− 0.492
Age	− 0.025	− 0.177	0.00	− 0.036	− 0.036

*R*^2^ = 0.061; ANOVA *P*< 0.001

## Discussion/conclusion

Japan is trying to cope with the aging of its population. The environment of medical care surrounding patients has greatly changed in recent years. In the present study, we correlated the medication of the elderly persons with xerostomia. Our results support the underlying situations wherein many elderly patients suffer from drug-induced xerostomia.

The predominant age distribution in this study was patients in their 70 s, and female patients were five times as predominant as male patients. It is reported that xerostomia patients in Sweden are predominantly in their 70 s, which is similar to Japan [12]. The morbidity of xerostomia is higher in female than in male patients. Although there is no difference in apparent sexual dimorphism between young and elderly patients, there are differences in xerostomia between elderly and young female patients [13]. The sex difference in xerostomia might be influenced by the sex hormones and differences in salivary gland tissues [14, 15]. We objectively evaluated hyposalivation using the Saxon test and moisture test.

The population of this study was xerostomia patients. That is, the group of patients with some kinds of xerostomia symptoms. But 65.3% of patients had negative results in the Saxon test, although all patients had symptoms of xerostomia. The cutoff score of the Saxon test was established based on the data of the SS patients [10, 16]. Because there are patients who were not diagnosed with SS in this present study, most patients were diagnosed as negative by the Saxon test. There were a lot of patients indicating equally negative even in a moisture test. In the present study, the percentage of patients who were diagnosed with SS was 8.1% (data not shown). In our clinic, we assessed the antibody SS-A and SS-B to screen for SS; however, the results of the screening test were not yet clear at the time of the first medical examination. Considering this data at the time of the first medical examination, SS was observed and patients were eventually diagnosed with SS. Additionally, these results lead us to conclude that symptoms and the results of diagnosis differ in several xerostomia patients. In some papers, “xerostomia” is interchangeably associated with “salivary gland hypofunction,” but in other papers, association between xerostomia and salivary gland hypofunction is still under investigation [17, 18]. Xerostomia is a subjective symptom, and salivary gland hypofunction is an objectively measured condition. In a systematic review, xerostomia prevalence ranged from 8 to 42%, while salivary gland hypofunction prevalence ranged from 12 to 47%. The prevalence of both conditions existing together is only approximately 2 to 6% [17, 19]. Our results also show that several patients experience xerostomia; however, during outpatient consultation, salivary gland hypofunction was not observed.

From such a background, patients do not suffer from xerostomia suddenly, and it is thought that xerostomia develops by the gradual accumulation of various factors. Using multiple regression analysis, we examined the factors affecting the Saxon test score for patients. Our results showed that age and sex significantly affected the Saxon test score; this data showed as same as the past study results [8]. The elderly patients showed harder hyposalivation than young patients except the factor of the medication. These results suggest that the number of drugs does not affect hyposalivation directly. In this study, it also shows that the number of the taking medicine per person increased with increasing age. It is thought that the number of drugs affects hyposalivation indirectly, as an ornamentation factor of the age. Xerostomia patients had several primary diseases, although it was subjectively assessed. High blood pressure and diabetes are considered predominant diseases in the elderly [20]. Diseases in the eyes and adnexa are the second most frequent diseases after high blood pressure, and xerostomia patients often experience dry eye syndrome. Hence, elderly patients with primary disease often experience xerostomia. Elderly individuals have a higher prevalence of disease, hence increasing the possibility of having to take several prescribed medications to treat their conditions. Correspondingly, taking several medicines is considered one likely cause of xerostomia, especially in elderly patients [21]. The xerostomia patients took an average of 5.58 ± 4.60 medicines, and most of these medicines include xerostomia as a possible side effect. Not only urinary retention drug reduces quantity of salivation by anticholinergic action, but also patients with urinary retention drug refrain from water intakes because they mind micturition. On the other hand, the antidepressant drugs intercept acetylcholine receptors and let anticholinergic action appear. In addition, many patients with antidepressant take anti-anxiety drug and sleeping drug, and they develop dry mouth symptom. With increasing age, the number of patients experiencing xerostomia increases. In particular, our results showed that patients taking more than 7–9 medications showed hyposalivation. Oral medicine, illness, and aging are considered causes of xerostomia. We investigated xerostomia patients with symptoms (such as xerostomia, glossalgia, and sticky saliva) during department consultation.

Moreover, interestingly, the young group took more medicines with xerostomia as a potential side effect than elderly patients. It is thought that young people are not as susceptible to the side effects medication [22]. It is possible that the young patients frequently take more drugs that include xerostomia as a possible side effect.

Representative drugs with xerostomia as the side effect are as follows: hypotensive agents, peptic ulcer therapeutic drugs, anti-anxiety drugs, and hypotensive agents^6^. Therefore, it is believed that medicines with xerostomia as the side effect more significantly affect the elderly patients than younger patients. The average number of drugs that the patients take is 5.58 ± 4.60 (range, 0–26), and the prices of these drugs were higher than that of the previous studies [23]. Each elderly xerostomia patient took an average of 6.8 ± 4.4 medicines. A total of 26.1% of patients in their 70 s took more than ten number of drugs. Considering the classification of medications according to their effect, the largest was peptic ulcer agent, followed in order by sedative, anxiolytic, psychoneurotic, and Chinese medicine. Several xerostomia therapeutic drugs were included in the Chinese medicine. A total of 40% of drugs were listed in a medical package insert that has xerostomia as a side effect. Although individually each side effect is not high, it is thought that xerostomia is observed because several drugs are being administered. Considering the classification according to the generic name, magnesium oxide, which is known as a laxative, is often used because prevalence of constipation is high in elderly individuals [24]. Considering that there are several hypertensive patients, Norvasc® (amlodipine besylate [generic name]) is administered as a therapeutic drug to treat high blood pressure. Symptoms of xerostomia were noted in hypertensive medicines with not only the calcium antagonists such as Norvasc® but also diuretic drugs such as Fluitran® (trichlormethiazide [generic name]) [25]. When patients take several drugs, even if these drugs have no side effect of xerostomia, xerostomia developed [6]. Our results show that xerostomia is a frequent side effect of taking several medicines. Nederfors et al. showed that there was a significant association between increasing xerostomia and the number of medications used [26]. IMS America’s National Prescription Audit detected 80.5% of drugs that xerostomia as the side effect [27]. Our data also revealed that taking several medicines causes xerostomia in the elderly.

It is believed that elderly patients taking several drugs often experience xerostomia symptoms. All patients experienced xerostomia symptoms in our study, but the percentage of patients who already received a prescribed xerostomia therapeutic drug was only 14.2%. Xerostomia therapeutic drugs have drug limitations and corresponding side effects [28, 29]. Moreover, the reason why several patients did not receive effective xerostomia treatment is that several patients only experienced symptoms of xerostomia but salivary gland hypofunction was not observed.

Our study has the following limitations. This study was not able to identify the drugs that caused xerostomia. This investigation is the crossing study that cut a point at one time. A follow-up survey and an intervention study are necessary to identify the real cause of xerostomia. However, studies regarding xerostomia are still developing, and it is important that we conduct research studies before conducting an intervention study. Next, it was difficult to digitize it about quality of the medicine in detail. We evaluated the quality of medication in reference to attached documents of each drug, but there were many drugs with unknown frequency in attached documents. There is various type of medicine that adjust water absorption or caused feeling thirsty. It is thought that new index to evaluate quality of the medicine objectively is necessary.

Elderly xerostomia patients are typically taking several numbers of medicines. Most of the medicines that are taken by these patients have xerostomia as the side effect. It is important that inspection and establishment of diagnosis should be performed properly because causes of xerostomia are multifactorial. For xerostomia patients, it is important that medicines should be continuously changed. It was revealed that many xerostomia patients took many drugs. The dentist confirms taking medicine not only to the xerostomia patients but also to the patients who do not notice xerostomia symptom.

The causes of xerostomia are unclear; hence, it requires comprehensive examination in several xerostomia patients. In conclusion, this study provides new and important information that will help in the development of xerostomia medical treatment.

App-Fig. 1. Hyposalivation of patients without mediation.

The graph shows the difference of hyposalivation between younger and elder patients without mediation. Score of Saxon test between < 65 years and ≥ 65 years patients without mediation. Saxon test scores < 2.00 (g/2 min) and ≥ 2.00 (g/2 min) are positive and negative for hyposalivation, respectively (A). Score of mouth moisture test between < 65 years and ≥ 65 years patients without mediation (B).

## Supplementary Information

Below is the link to the electronic supplementary material.

Supplementary file1 (PPTX 27 KB)
