# Supplementary material for: Analysis of medication-induced xerostomia in elderly Japanese patients
Source: Clin Oral Investig. 2021 Sep 28;26(2):2021–9. doi: 10.1007/s00784-021-04182-2 (PMC8816514; doi:10.1007/s00784-021-04182-2)
Supplement: Supplementary file 1 — Supplementary file1 (PPTX 27 KB) [file 784_2021_4182_MOESM1_ESM.pptx]

## Slide 1
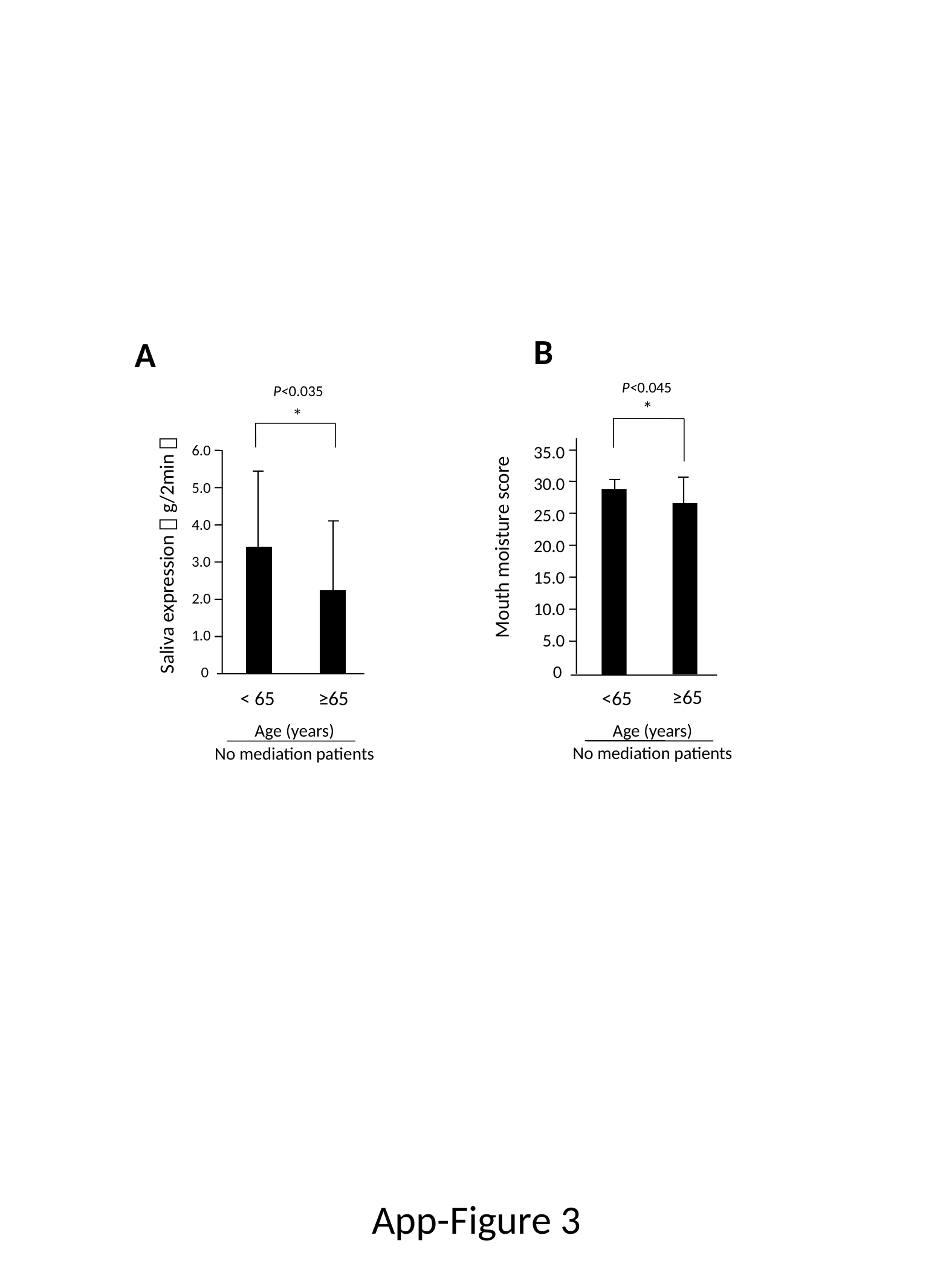

B
A
P<0.035
*
6.0
5.0
4.0
3.0
2.0
1.0
0
< 65
≥65
P<0.045
*
35.0
30.0
25.0
Mouth moisture score
Saliva expression（g/2min）
20.0
15.0
10.0
5.0
0
≥65
<65
Age (years)
No mediation patients
Age (years)
No mediation patients
App-Figure 3
